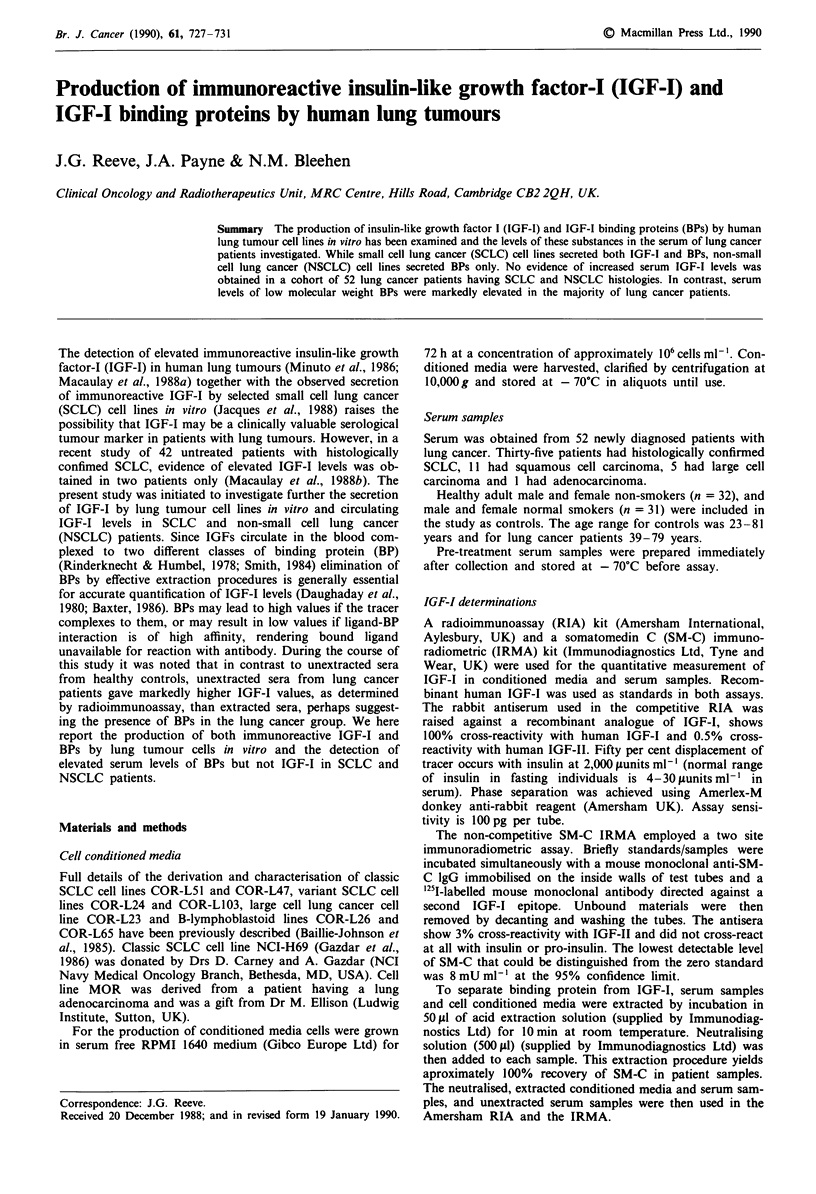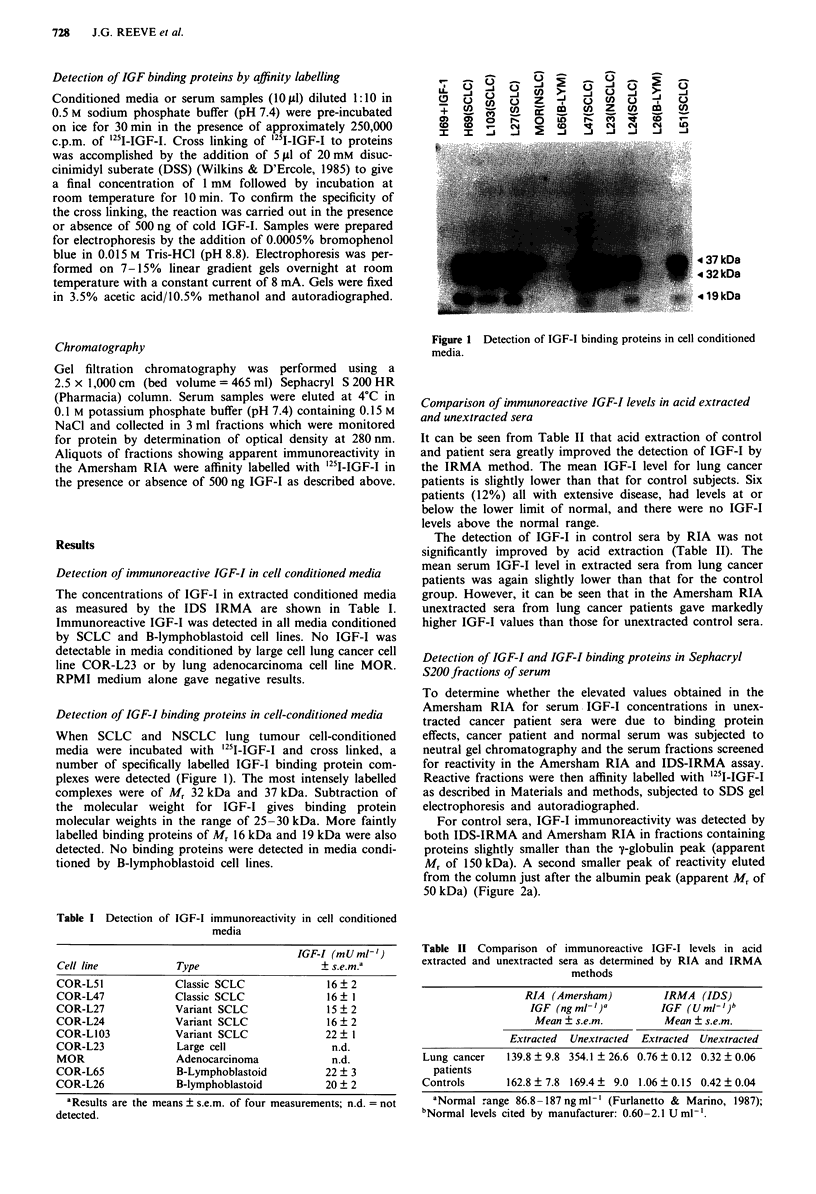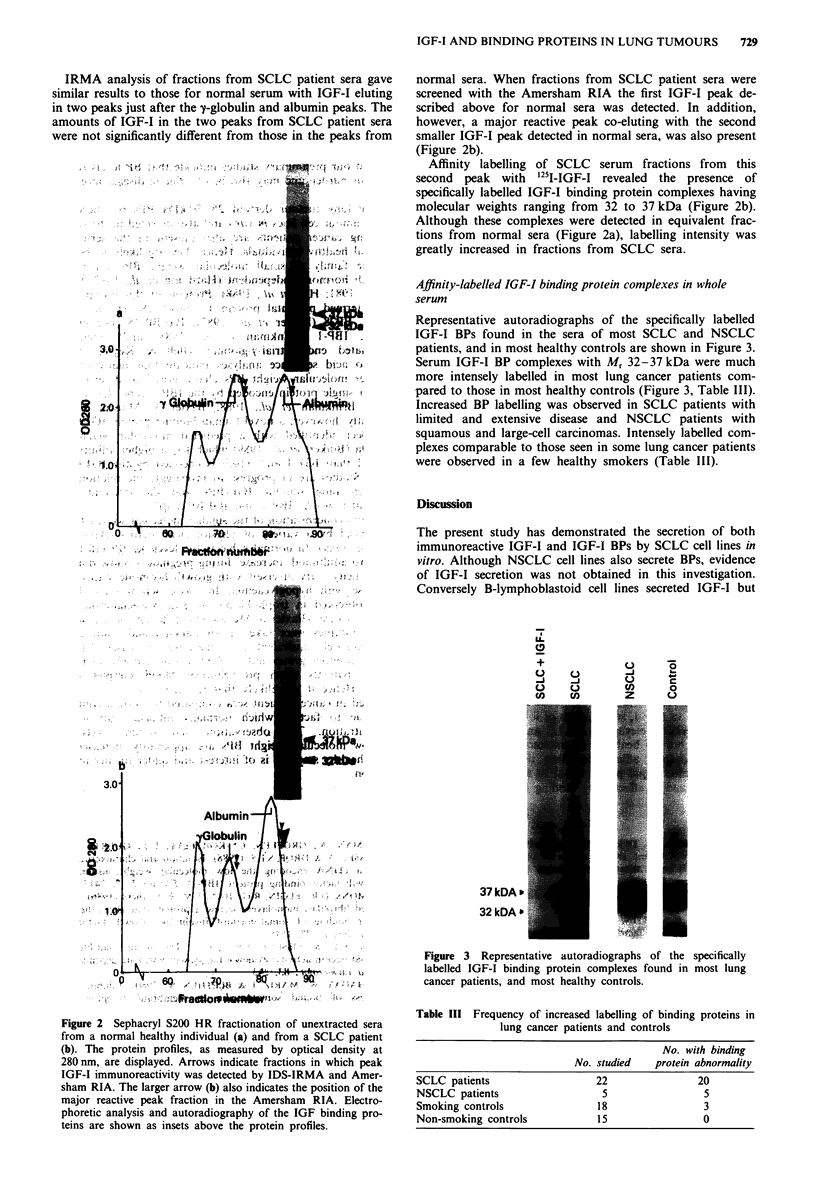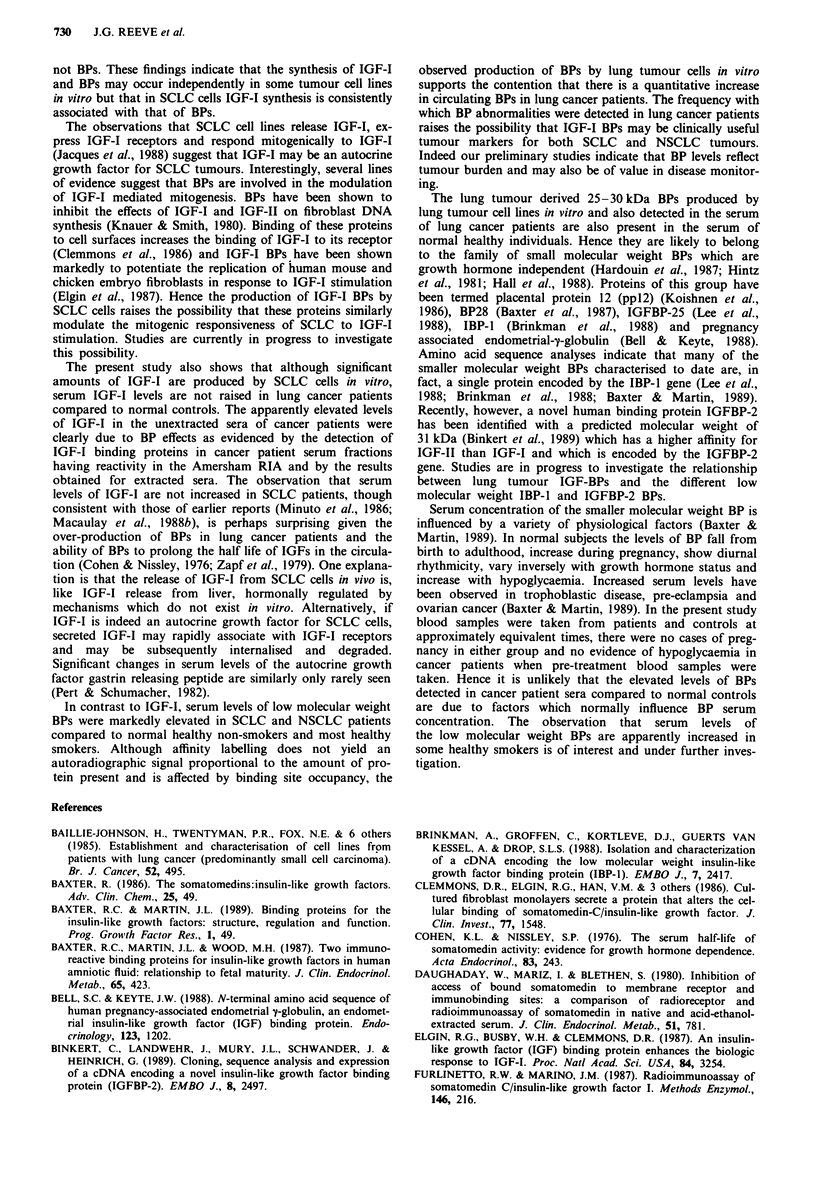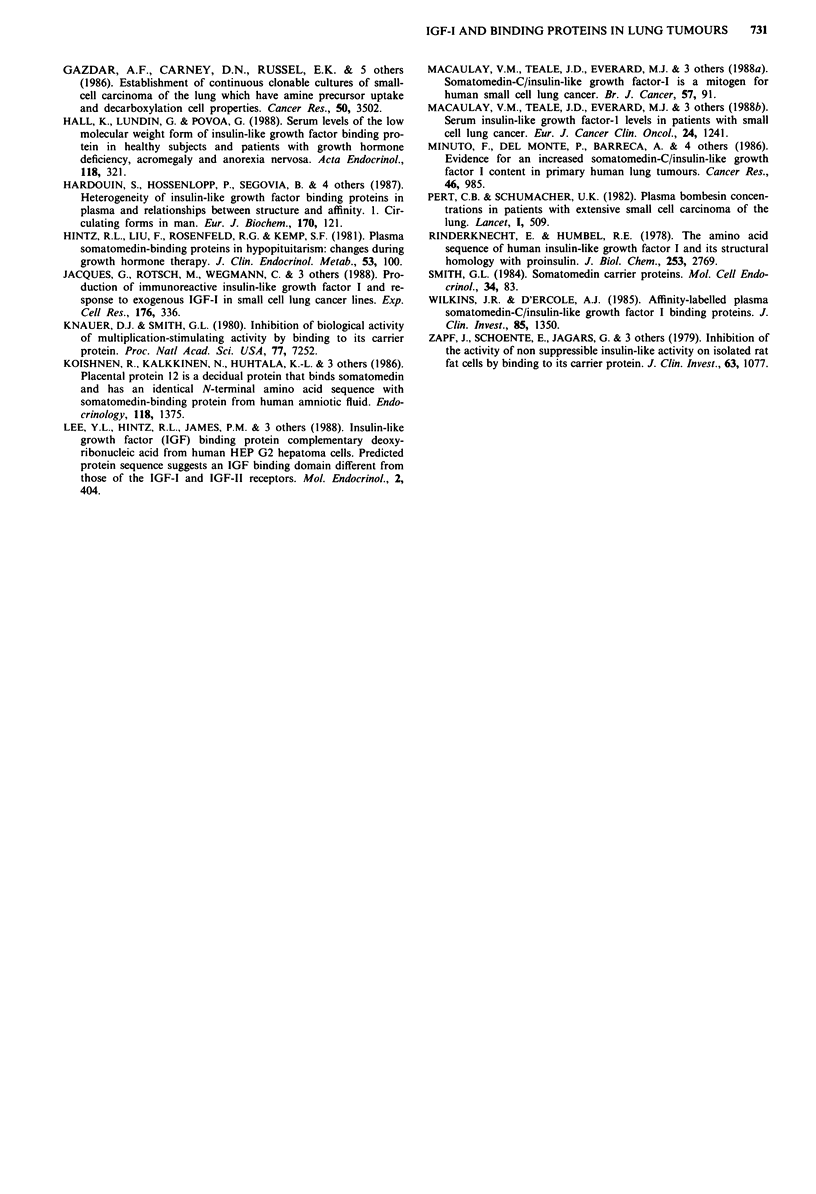# Production of immunoreactive insulin-like growth factor-I (IGF-I) and IGF-I binding proteins by human lung tumours.

**DOI:** 10.1038/bjc.1990.163

**Published:** 1990-05

**Authors:** J. G. Reeve, J. A. Payne, N. M. Bleehen

**Affiliations:** Clinical Oncology and Radiotherapeutics Unit, MRC Centre, Cambridge, UK.

## Abstract

**Images:**


*Br. J. Cancer* (1990), **61**, 727–731 © Macmillan Press Ltd., 1990

### Production of immunoreactive insulin-like growth factor-I (IGF-I) and IGF-I binding proteins by human lung tumours

J.G. Reeve, J.A. Payne & N.M. Bleehen

*Clinical Oncology and Radiotherapeutics Unit, MRC Centre, Hills Road, Cambridge CB2 2QH, UK.*

**Summary** The production of insulin-like growth factor I (IGF-I) and IGF-I binding proteins (BPs) by human lung tumour cell lines *in vitro* has been examined and the levels of these substances in the serum of lung cancer patients investigated. While small cell lung cancer (SCLC) cell lines secreted both IGF-I and BPs, non-small cell lung cancer (NSCLC) cell lines secreted BPs only. No evidence of increased serum IGF-I levels was obtained in a cohort of 52 lung cancer patients having SCLC and NSCLC histologies. In contrast, serum levels of low molecular weight BPs were markedly elevated in the majority of lung cancer patients.

The detection of elevated immunoreactive insulin-like growth factor-I (IGF-I) in human lung tumours (Minuto *et al.*, 1986; Macaulay *et al.*, 1988*a*) together with the observed secretion of immunoreactive IGF-I by selected small cell lung cancer (SCLC) cell lines *in vitro* (Jacques *et al.*, 1988) raises the possibility that IGF-I may be a clinically valuable serological tumour marker in patients with lung tumours. However, in a recent study of 42 untreated patients with histologically confimed SCLC, evidence of elevated IGF-I levels was obtained in two patients only (Macaulay *et al.*, 1988*b*). The present study was initiated to investigate further the secretion of IGF-I by lung tumour cell lines *in vitro* and circulating IGF-I levels in SCLC and non-small cell lung cancer (NSCLC) patients. Since IGFs circulate in the blood complexed to two different classes of binding protein (BP) (Rinderknecht & Humbel, 1978; Smith, 1984) elimination of BPs by effective extraction procedures is generally essential for accurate quantification of IGF-I levels (Daughaday *et al.*, 1980; Baxter, 1986). BPs may lead to high values if the tracer complexes to them, or may result in low values if ligand-BP interaction is of high affinity, rendering bound ligand unavailable for reaction with antibody. During the course of this study it was noted that in contrast to unextracted sera from healthy controls, unextracted sera from lung cancer patients gave markedly higher IGF-I values, as determined by radioimmunoassay, than extracted sera, perhaps suggesting the presence of BPs in the lung cancer group. We here report the production of both immunoreactive IGF-I and BPs by lung tumour cells *in vitro* and the detection of elevated serum levels of BPs but not IGF-I in SCLC and NSCLC patients.

### Materials and methods

### *Cell conditioned media*

Full details of the derivation and characterisation of classic SCLC cell lines COR-L51 and COR-L47, variant SCLC cell lines COR-L24 and COR-L103, large cell lung cancer cell line COR-L23 and B-lymphoblastoid lines COR-L26 and COR-L65 have been previously described (Baillie-Johnson *et al.*, 1985). Classic SCLC cell line NCI-H69 (Gazdar *et al.*, 1986) was donated by Drs D. Carney and A. Gazdar (NCI Navy Medical Oncology Branch, Bethesda, MD, USA). Cell line MOR was derived from a patient having a lung adenocarcinoma and was a gift from Dr M. Ellison (Ludwig Institute, Sutton, UK).

For the production of conditioned media cells were grown in serum free RPMI 1640 medium (Gibco Europe Ltd) for 72 h at a concentration of approximately $10^6$ cells $ml^{-1}$. Conditioned media were harvested, clarified by centrifugation at 10,000 *g* and stored at − 70°C in aliquots until use.

### *Serum samples*

Serum was obtained from 52 newly diagnosed patients with lung cancer. Thirty-five patients had histologically confirmed SCLC, 11 had squamous cell carcinoma, 5 had large cell carcinoma and 1 had adenocarcinoma.

Healthy adult male and female non-smokers ($n = 32$), and male and female normal smokers ($n = 31$) were included in the study as controls. The age range for controls was 23–81 years and for lung cancer patients 39–79 years.

Pre-treatment serum samples were prepared immediately after collection and stored at − 70°C before assay.

### *IGF-I determinations*

A radioimmunoassay (RIA) kit (Amersham International, Aylesbury, UK) and a somatomedin C (SM-C) immunoradiometric (IRMA) kit (Immunodiagnostics Ltd, Tyne and Wear, UK) were used for the quantitative measurement of IGF-I in conditioned media and serum samples. Recombinant human IGF-I was used as standards in both assays. The rabbit antiserum used in the competitive RIA was raised against a recombinant analogue of IGF-I, shows 100% cross-reactivity with human IGF-I and 0.5% cross-reactivity with human IGF-II. Fifty per cent displacement of tracer occurs with insulin at 2,000 μunits $ml^{-1}$ (normal range of insulin in fasting individuals is 4–30 μunits $ml^{-1}$ in serum). Phase separation was achieved using Amerlex-M donkey anti-rabbit reagent (Amersham UK). Assay sensitivity is 100 pg per tube.

The non-competitive SM-C IRMA employed a two site immunoradiometric assay. Briefly standards/samples were incubated simultaneously with a mouse monoclonal anti-SM-C IgG immobilised on the inside walls of test tubes and a $^{125}$I-labelled mouse monoclonal antibody directed against a second IGF-I epitope. Unbound materials were then removed by decanting and washing the tubes. The antisera show 3% cross-reactivity with IGF-II and did not cross-react at all with insulin or pro-insulin. The lowest detectable level of SM-C that could be distinguished from the zero standard was 8 mU $ml^{-1}$ at the 95% confidence limit.

To separate binding protein from IGF-I, serum samples and cell conditioned media were extracted by incubation in 50 μl of acid extraction solution (supplied by Immunodiagnostics Ltd) for 10 min at room temperature. Neutralising solution (500 μl) (supplied by Immunodiagnostics Ltd) was then added to each sample. This extraction procedure yields aproximately 100% recovery of SM-C in patient samples. The neutralised, extracted conditioned media and serum samples, and unextracted serum samples were then used in the Amersham RIA and the IRMA.

Correspondence: J.G. Reeve.
Received 20 December 1988; and in revised form 19 January 1990.

*Detection of IGF binding proteins by affinity labelling*

Conditioned media or serum samples (10 µl) diluted 1:10 in 0.5 M sodium phosphate buffer (pH 7.4) were pre-incubated on ice for 30 min in the presence of approximately 250,000 c.p.m. of $^{125}$I-IGF-I. Cross linking of $^{125}$I-IGF-I to proteins was accomplished by the addition of 5 µl of 20 mM disuccinimidyl suberate (DSS) (Wilkins & D'Ercole, 1985) to give a final concentration of 1 mM followed by incubation at room temperature for 10 min. To confirm the specificity of the cross linking, the reaction was carried out in the presence or absence of 500 ng of cold IGF-I. Samples were prepared for electrophoresis by the addition of 0.0005% bromophenol blue in 0.015 M Tris-HCl (pH 8.8). Electrophoresis was performed on 7–15% linear gradient gels overnight at room temperature with a constant current of 8 mA. Gels were fixed in 3.5% acetic acid/10.5% methanol and autoradiographed.

*Chromatography*

Gel filtration chromatography was performed using a 2.5 × 1,000 cm (bed volume = 465 ml) Sephacryl S 200 HR (Pharmacia) column. Serum samples were eluted at 4°C in 0.1 M potassium phosphate buffer (pH 7.4) containing 0.15 M NaCl and collected in 3 ml fractions which were monitored for protein by determination of optical density at 280 nm. Aliquots of fractions showing apparent immunoreactivity in the Amersham RIA were affinity labelled with $^{125}$I-IGF-I in the presence or absence of 500 ng IGF-I as described above.

### Results

*Detection of immunoreactive IGF-I in cell conditioned media*

The concentrations of IGF-I in extracted conditioned media as measured by the IDS IRMA are shown in Table I. Immunoreactive IGF-I was detected in all media conditioned by SCLC and B-lymphoblastoid cell lines. No IGF-I was detectable in media conditioned by large cell lung cancer cell line COR-L23 or by lung adenocarcinoma cell line MOR. RPMI medium alone gave negative results.

*Detection of IGF-I binding proteins in cell-conditioned media*

When SCLC and NSCLC lung tumour cell-conditioned media were incubated with $^{125}$I-IGF-I and cross linked, a number of specifically labelled IGF-I binding protein complexes were detected (Figure 1). The most intensely labelled complexes were of $M_r$ 32 kDa and 37 kDa. Subtraction of the molecular weight for IGF-I gives binding protein molecular weights in the range of 25–30 kDa. More faintly labelled binding proteins of $M_r$ 16 kDa and 19 kDa were also detected. No binding proteins were detected in media conditioned by B-lymphoblastoid cell lines.

**Table I** Detection of IGF-I immunoreactivity in cell conditioned media

| *Cell line* | *Type* | *IGF-I (mU ml$^{-1}$) ± s.e.m.*[a] |
|---|---|---|
| COR-L51 | Classic SCLC | 16 ± 2 |
| COR-L47 | Classic SCLC | 16 ± 1 |
| COR-L27 | Variant SCLC | 15 ± 2 |
| COR-L24 | Variant SCLC | 16 ± 2 |
| COR-L103 | Variant SCLC | 22 ± 1 |
| COR-L23 | Large cell | n.d. |
| MOR | Adenocarcinoma | n.d. |
| COR-L65 | B-Lymphoblastoid | 22 ± 3 |
| COR-L26 | B-lymphoblastoid | 20 ± 2 |

[a]Results are the means ± s.e.m. of four measurements; n.d. = not detected.

**Figure 1** Detection of IGF-I binding proteins in cell conditioned media.

*Comparison of immunoreactive IGF-I levels in acid extracted and unextracted sera*

It can be seen from Table II that acid extraction of control and patient sera greatly improved the detection of IGF-I by the IRMA method. The mean IGF-I level for lung cancer patients is slightly lower than that for control subjects. Six patients (12%) all with extensive disease, had levels at or below the lower limit of normal, and there were no IGF-I levels above the normal range.

The detection of IGF-I in control sera by RIA was not significantly improved by acid extraction (Table II). The mean serum IGF-I level in extracted sera from lung cancer patients was again slightly lower than that for the control group. However, it can be seen that in the Amersham RIA unextracted sera from lung cancer patients gave markedly higher IGF-I values than those for unextracted control sera.

*Detection of IGF-I and IGF-I binding proteins in Sephacryl S200 fractions of serum*

To determine whether the elevated values obtained in the Amersham RIA for serum IGF-I concentrations in unextracted cancer patient sera were due to binding protein effects, cancer patient and normal serum was subjected to neutral gel chromatography and the serum fractions screened for reactivity in the Amersham RIA and IDS-IRMA assay. Reactive fractions were then affinity labelled with $^{125}$I-IGF-I as described in Materials and methods, subjected to SDS gel electrophoresis and autoradiographed.

For control sera, IGF-I immunoreactivity was detected by both IDS-IRMA and Amersham RIA in fractions containing proteins slightly smaller than the γ-globulin peak (apparent $M_r$ of 150 kDa). A second smaller peak of reactivity eluted from the column just after the albumin peak (apparent $M_r$ of 50 kDa) (Figure 2a).

**Table II** Comparison of immunoreactive IGF-I levels in acid extracted and unextracted sera as determined by RIA and IRMA methods

| | *RIA (Amersham) IGF (ng ml$^{-1}$)*[a] *Mean ± s.e.m.* | | *IRMA (IDS) IGF (U ml$^{-1}$)*[b] *Mean ± s.e.m.* | |
|---|---|---|---|---|
| | *Extracted* | *Unextracted* | *Extracted* | *Unextracted* |
| Lung cancer patients | 139.8 ± 9.8 | 354.1 ± 26.6 | 0.76 ± 0.12 | 0.32 ± 0.06 |
| Controls | 162.8 ± 7.8 | 169.4 ± 9.0 | 1.06 ± 0.15 | 0.42 ± 0.04 |

[a]Normal range 86.8–187 ng ml$^{-1}$ (Furlanetto & Marino, 1987); [b]Normal levels cited by manufacturer: 0.60–2.1 U ml$^{-1}$.

IRMA analysis of fractions from SCLC patient sera gave similar results to those for normal serum with IGF-I eluting in two peaks just after the γ-globulin and albumin peaks. The amounts of IGF-I in the two peaks from SCLC patient sera were not significantly different from those in the peaks from normal sera. When fractions from SCLC patient sera were screened with the Amersham RIA the first IGF-I peak described above for normal sera was detected. In addition, however, a major reactive peak co-eluting with the second smaller IGF-I peak detected in normal sera, was also present (Figure 2b).

Affinity labelling of SCLC serum fractions from this second peak with $^{125}$I-IGF-I revealed the presence of specifically labelled IGF-I binding protein complexes having molecular weights ranging from 32 to 37 kDa (Figure 2b). Although these complexes were detected in equivalent fractions from normal sera (Figure 2a), labelling intensity was greatly increased in fractions from SCLC sera.

**Figure 2** Sephacryl S200 HR fractionation of unextracted sera from a normal healthy individual (**a**) and from a SCLC patient (**b**). The protein profiles, as measured by optical density at 280 nm, are displayed. Arrows indicate fractions in which peak IGF-I immunoreactivity was detected by IDS-IRMA and Amersham RIA. The larger arrow (**b**) also indicates the position of the major reactive peak fraction in the Amersham RIA. Electrophoretic analysis and autoradiography of the IGF binding proteins are shown as insets above the protein profiles.

### *Affinity-labelled IGF-I binding protein complexes in whole serum*

Representative autoradiographs of the specifically labelled IGF-I BPs found in the sera of most SCLC and NSCLC patients, and in most healthy controls are shown in Figure 3. Serum IGF-I BP complexes with $M_r$ 32–37 kDa were much more intensely labelled in most lung cancer patients compared to those in most healthy controls (Figure 3, Table III). Increased BP labelling was observed in SCLC patients with limited and extensive disease and NSCLC patients with squamous and large-cell carcinomas. Intensely labelled complexes comparable to those seen in some lung cancer patients were observed in a few healthy smokers (Table III).

### Discussion

The present study has demonstrated the secretion of both immunoreactive IGF-I and IGF-I BPs by SCLC cell lines *in vitro*. Although NSCLC cell lines also secrete BPs, evidence of IGF-I secretion was not obtained in this investigation. Conversely B-lymphoblastoid cell lines secreted IGF-I but

**Figure 3** Representative autoradiographs of the specifically labelled IGF-I binding protein complexes found in most lung cancer patients, and most healthy controls.

**Table III** Frequency of increased labelling of binding proteins in lung cancer patients and controls

| | *No. studied* | *No. with binding protein abnormality* |
|---|---|---|
| SCLC patients | 22 | 20 |
| NSCLC patients | 5 | 5 |
| Smoking controls | 18 | 3 |
| Non-smoking controls | 15 | 0 |

not BPs. These findings indicate that the synthesis of IGF-I and BPs may occur independently in some tumour cell lines *in vitro* but that in SCLC cells IGF-I synthesis is consistently associated with that of BPs.

The observations that SCLC cell lines release IGF-I, express IGF-I receptors and respond mitogenically to IGF-I (Jacques *et al.*, 1988) suggest that IGF-I may be an autocrine growth factor for SCLC tumours. Interestingly, several lines of evidence suggest that BPs are involved in the modulation of IGF-I mediated mitogenesis. BPs have been shown to inhibit the effects of IGF-I and IGF-II on fibroblast DNA synthesis (Knauer & Smith, 1980). Binding of these proteins to cell surfaces increases the binding of IGF-I to its receptor (Clemmons *et al.*, 1986) and IGF-I BPs have been shown markedly to potentiate the replication of human mouse and chicken embryo fibroblasts in response to IGF-I stimulation (Elgin *et al.*, 1987). Hence the production of IGF-I BPs by SCLC cells raises the possibility that these proteins similarly modulate the mitogenic responsiveness of SCLC to IGF-I stimulation. Studies are currently in progress to investigate this possibility.

The present study also shows that although significant amounts of IGF-I are produced by SCLC cells *in vitro*, serum IGF-I levels are not raised in lung cancer patients compared to normal controls. The apparently elevated levels of IGF-I in the unextracted sera of cancer patients were clearly due to BP effects as evidenced by the detection of IGF-I binding proteins in cancer patient serum fractions having reactivity in the Amersham RIA and by the results obtained for extracted sera. The observation that serum levels of IGF-I are not increased in SCLC patients, though consistent with those of earlier reports (Minuto *et al.*, 1986; Macaulay *et al.*, 1988*b*), is perhaps surprising given the over-production of BPs in lung cancer patients and the ability of BPs to prolong the half life of IGFs in the circulation (Cohen & Nissley, 1976; Zapf *et al.*, 1979). One explanation is that the release of IGF-I from SCLC cells *in vivo* is, like IGF-I release from liver, hormonally regulated by mechanisms which do not exist *in vitro*. Alternatively, if IGF-I is indeed an autocrine growth factor for SCLC cells, secreted IGF-I may rapidly associate with IGF-I receptors and may be subsequently internalised and degraded. Significant changes in serum levels of the autocrine growth factor gastrin releasing peptide are similarly only rarely seen (Pert & Schumacher, 1982).

In contrast to IGF-I, serum levels of low molecular weight BPs were markedly elevated in SCLC and NSCLC patients compared to normal healthy non-smokers and most healthy smokers. Although affinity labelling does not yield an autoradiographic signal proportional to the amount of protein present and is affected by binding site occupancy, the observed production of BPs by lung tumour cells *in vitro* supports the contention that there is a quantitative increase in circulating BPs in lung cancer patients. The frequency with which BP abnormalities were detected in lung cancer patients raises the possibility that IGF-I BPs may be clinically useful tumour markers for both SCLC and NSCLC tumours. Indeed our preliminary studies indicate that BP levels reflect tumour burden and may also be of value in disease monitoring.

The lung tumour derived 25–30 kDa BPs produced by lung tumour cell lines *in vitro* and also detected in the serum of lung cancer patients are also present in the serum of normal healthy individuals. Hence they are likely to belong to the family of small molecular weight BPs which are growth hormone independent (Hardouin *et al.*, 1987; Hintz *et al.*, 1981; Hall *et al.*, 1988). Proteins of this group have been termed placental protein 12 (pp12) (Koishnen *et al.*, 1986), BP28 (Baxter *et al.*, 1987), IGFBP-25 (Lee *et al.*, 1988), IBP-1 (Brinkman *et al.*, 1988) and pregnancy associated endometrial-γ-globulin (Bell & Keyte, 1988). Amino acid sequence analyses indicate that many of the smaller molecular weight BPs characterised to date are, in fact, a single protein encoded by the IBP-1 gene (Lee *et al.*, 1988; Brinkman *et al.*, 1988; Baxter & Martin, 1989). Recently, however, a novel human binding protein IGFBP-2 has been identified with a predicted molecular weight of 31 kDa (Binkert *et al.*, 1989) which has a higher affinity for IGF-II than IGF-I and which is encoded by the IGFBP-2 gene. Studies are in progress to investigate the relationship between lung tumour IGF-BPs and the different low molecular weight IBP-1 and IGFBP-2 BPs.

Serum concentration of the smaller molecular weight BP is influenced by a variety of physiological factors (Baxter & Martin, 1989). In normal subjects the levels of BP fall from birth to adulthood, increase during pregnancy, show diurnal rhythmicity, vary inversely with growth hormone status and increase with hypoglycaemia. Increased serum levels have been observed in trophoblastic disease, pre-eclampsia and ovarian cancer (Baxter & Martin, 1989). In the present study blood samples were taken from patients and controls at approximately equivalent times, there were no cases of pregnancy in either group and no evidence of hypoglycaemia in cancer patients when pre-treatment blood samples were taken. Hence it is unlikely that the elevated levels of BPs detected in cancer patient sera compared to normal controls are due to factors which normally influence BP serum concentration. The observation that serum levels of the low molecular weight BPs are apparently increased in some healthy smokers is of interest and under further investigation.

### References

BAILLIE-JOHNSON, H., TWENTYMAN, P.R., FOX, N.E. & 6 others (1985). Establishment and characterisation of cell lines from patients with lung cancer (predominantly small cell carcinoma). *Br. J. Cancer*, **52**, 495.

BAXTER, R. (1986). The somatomedins:insulin-like growth factors. *Adv. Clin. Chem.*, **25**, 49.

BAXTER, R.C. & MARTIN, J.L. (1989). Binding proteins for the insulin-like growth factors: structure, regulation and function. *Prog. Growth Factor Res.*, **1**, 49.

BAXTER, R.C., MARTIN, J.L. & WOOD, M.H. (1987). Two immunoreactive binding proteins for insulin-like growth factors in human amniotic fluid: relationship to fetal maturity. *J. Clin. Endocrinol. Metab.*, **65**, 423.

BELL, S.C. & KEYTE, J.W. (1988). *N*-terminal amino acid sequence of human pregnancy-associated endometrial γ-globulin, an endometrial insulin-like growth factor (IGF) binding protein. *Endocrinology*, **123**, 1202.

BINKERT, C., LANDWEHR, J., MURY, J.L., SCHWANDER, J. & HEINRICH, G. (1989). Cloning, sequence analysis and expression of a cDNA encoding a novel insulin-like growth factor binding protein (IGFBP-2). *EMBO J.*, **8**, 2497.

BRINKMAN, A., GROFFEN, C., KORTLEVE, D.J., GUERTS VAN KESSEL, A. & DROP, S.L.S. (1988). Isolation and characterization of a cDNA encoding the low molecular weight insulin-like growth factor binding protein (IBP-1). *EMBO J.*, **7**, 2417.

CLEMMONS, D.R., ELGIN, R.G., HAN, V.M. & 3 others (1986). Cultured fibroblast monolayers secrete a protein that alters the cellular binding of somatomedin-C/insulin-like growth factor. *J. Clin. Invest.*, **77**, 1548.

COHEN, K.L. & NISSLEY, S.P. (1976). The serum half-life of somatomedin activity: evidence for growth hormone dependence. *Acta Endocrinol.*, **83**, 243.

DAUGHADAY, W., MARIZ, I. & BLETHEN, S. (1980). Inhibition of access of bound somatomedin to membrane receptor and immunobinding sites: a comparison of radioreceptor and radioimmunoassay of somatomedin in native and acid-ethanol-extracted serum. *J. Clin. Endocrinol. Metab.*, **51**, 781.

ELGIN, R.G., BUSBY, W.H. & CLEMMONS, D.R. (1987). An insulin-like growth factor (IGF) binding protein enhances the biologic response to IGF-I. *Proc. Natl Acad. Sci. USA*, **84**, 3254.

FURLINETTO, R.W. & MARINO, J.M. (1987). Radioimmunoassay of somatomedin C/insulin-like growth factor I. *Methods Enzymol.*, **146**, 216.

GAZDAR, A.F., CARNEY, D.N., RUSSEL, E.K. & 5 others (1986). Establishment of continuous clonable cultures of small-cell carcinoma of the lung which have amine precursor uptake and decarboxylation cell properties. *Cancer Res.*, **50**, 3502.

HALL, K., LUNDIN, G. & POVOA, G. (1988). Serum levels of the low molecular weight form of insulin-like growth factor binding protein in healthy subjects and patients with growth hormone deficiency, acromegaly and anorexia nervosa. *Acta Endocrinol.*, **118**, 321.

HARDOUIN, S., HOSSENLOPP, P., SEGOVIA, B. & 4 others (1987). Heterogeneity of insulin-like growth factor binding proteins in plasma and relationships between structure and affinity. 1. Circulating forms in man. *Eur. J. Biochem.*, **170**, 121.

HINTZ, R.L., LIU, F., ROSENFELD, R.G. & KEMP, S.F. (1981). Plasma somatomedin-binding proteins in hypopituitarism: changes during growth hormone therapy. *J. Clin. Endocrinol. Metab.*, **53**, 100.

JACQUES, G., ROTSCH, M., WEGMANN, C. & 3 others (1988). Production of immunoreactive insulin-like growth factor I and response to exogenous IGF-I in small cell lung cancer lines. *Exp. Cell Res.*, **176**, 336.

KNAUER, D.J. & SMITH, G.L. (1980). Inhibition of biological activity of multiplication-stimulating activity by binding to its carrier protein. *Proc. Natl Acad. Sci. USA*, **77**, 7252.

KOISHNEN, R., KALKKINEN, N., HUHTALA, K.-L. & 3 others (1986). Placental protein 12 is a decidual protein that binds somatomedin and has an identical *N*-terminal amino acid sequence with somatomedin-binding protein from human amniotic fluid. *Endocrinology*, **118**, 1375.

LEE, Y.L., HINTZ, R.L., JAMES, P.M. & 3 others (1988). Insulin-like growth factor (IGF) binding protein complementary deoxyribonucleic acid from human HEP G2 hepatoma cells. Predicted protein sequence suggests an IGF binding domain different from those of the IGF-I and IGF-II receptors. *Mol. Endocrinol.*, **2**, 404.

MACAULAY, V.M., TEALE, J.D., EVERARD, M.J. & 3 others (1988*a*). Somatomedin-C/insulin-like growth factor-I is a mitogen for human small cell lung cancer. *Br. J. Cancer*, **57**, 91.

MACAULAY, V.M., TEALE, J.D., EVERARD, M.J. & 3 others (1988*b*). Serum insulin-like growth factor-1 levels in patients with small cell lung cancer. *Eur. J. Cancer Clin. Oncol.*, **24**, 1241.

MINUTO, F., DEL MONTE, P., BARRECA, A. & 4 others (1986). Evidence for an increased somatomedin-C/insulin-like growth factor I content in primary human lung tumours. *Cancer Res.*, **46**, 985.

PERT, C.B. & SCHUMACHER, U.K. (1982). Plasma bombesin concentrations in patients with extensive small cell carcinoma of the lung. *Lancet*, **I**, 509.

RINDERKNECHT, E. & HUMBEL, R.E. (1978). The amino acid sequence of human insulin-like growth factor I and its structural homology with proinsulin. *J. Biol. Chem.*, **253**, 2769.

SMITH, G.L. (1984). Somatomedin carrier proteins. *Mol. Cell Endocrinol.*, **34**, 83.

WILKINS, J.R. & D'ERCOLE, A.J. (1985). Affinity-labelled plasma somatomedin-C/insulin-like growth factor I binding proteins. *J. Clin. Invest.*, **85**, 1350.

ZAPF, J., SCHOENTE, E., JAGARS, G. & 3 others (1979). Inhibition of the activity of non suppressible insulin-like activity on isolated rat fat cells by binding to its carrier protein. *J. Clin. Invest.*, **63**, 1077.